# Aging Impacts Basic Auditory and Timing Processes

**DOI:** 10.1111/ejn.70031

**Published:** 2025-03-03

**Authors:** Antonio Criscuolo, Michael Schwartze, Leonardo Bonetti, Sonja A. Kotz

**Affiliations:** ^1^ Department of Neuropsychology & Psychopharmacology, Faculty of Psychology and Neuroscience Maastricht University Maastricht the Netherlands; ^2^ Center for Music in the Brain, Department of Clinical Medicine Aarhus University & the Royal Academy of Music Aalborg Denmark; ^3^ Centre for Eudaimonia and Human Flourishing, Linacre College University of Oxford Oxford UK; ^4^ Department of Psychiatry University of Oxford Oxford UK; ^5^ Department of Neuropsychology Max Planck Institute for Human Cognitive and Brain Sciences Leipzig Germany

**Keywords:** aging, audition, EEG, oscillations, timing

## Abstract

Deterioration in the peripheral and central auditory systems is common in older adults and often leads to hearing and speech comprehension difficulties. Even when hearing remains intact, electrophysiological data of older adults frequently exhibit altered neural responses along the auditory pathway, reflected in variability in phase alignment of neural activity to speech sound onsets. However, it remains unclear whether challenges in speech processing in aging stem from more fundamental deficits in auditory and timing processes. Here, we investigated if and how aging individuals encoded temporal regularities in isochronous auditory sequences presented at 1.5Hz, and if they employed adaptive mechanisms of neural phase alignment in anticipation of next sound onsets. We recorded EEG in older and young individuals listening to simple isochronous tone sequences. We show that aging individuals displayed larger event‐related neural responses, an increased 1/F slope, but reduced phase‐coherence at the stimulation frequency (1.5Hz) and a reduced slope of phase‐coherence over time in the delta and theta frequency‐bands. These observations suggest altered top‐down modulatory inhibition when processing repeated and predictable sounds in a sequence and altered mechanisms of continuous phase‐alignment to expected sound onsets in aging. Given that deteriorations in these basic timing capacities may affect other higher‐order cognitive processes (e.g., attention, perception, and action), these results underscore the need for future research examining the link between basic timing abilities and general cognition across the lifespan.

## Graphical abstract

We investigated if and how aging impacts basic timing processes in audition. Healthy young and older individuals listened to isochronous auditory sequences while we recorded their EEG. Analyses showed increased event‐related responses and 1/F, but reduced phase coherence in older individuals. These observations suggest that aging alters the way we process predictable sounds and temporal regularities, and motivates future research on the link with speech comprehension deficits.

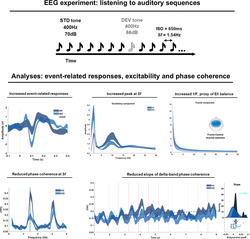

## 1 Introduction

“Wait, what? Can you repeat it?”

A cascade of biochemical, neuro‐functional, and ‐anatomical changes takes place in aging. Deteriorations in the peripheral (e.g., loss of hair, ganglion and/or striatal cells) and central auditory systems (Gates and Mills 2005; Ouda, Profant, and Syka 2015) are particularly common and typically lead to a decline in auditory processing capacity (Bidelman et al. 2014, 2019; Bidelman and Alain 2015; Lai, Alain, and Bidelman 2023). However, structural brain changes often extend more broadly, and include widespread reductions in gray and white matter volume across the brain (Raz and Daugherty 2018), as well as in cortico‐subcortical connectivity (Bostan and Strick 2018). Moreover, modifications within striatal‐frontal networks (Buckner 2004), under‐recruitment of the cerebellum during challenging cognitive tasks (Bernard et al. 2020), and alterations in cerebellum‐basal ganglia connectivity (Hausman et al. 2020) have been linked to diminished cognitive control (Spreng and Turner 2021) and a variety of motor and cognitive deficits (Hausman et al. 2020). However, there is significant heterogeneity in the trajectories of neurocognitive and structural decline, stemming from substantial inter‐individual variability in risk and modulating factors (Reuter‐Lorenz and Park 2014). Furthermore, there exists variability in the capacity to compensate for cognitive decline by recruiting additional neural resources and/or adopting compensatory cognitive strategies (Reuter‐Lorenz and Park 2014). For example, despite inevitable hearing loss (Gates and Mills 2005; Ouda, Profant, and Syka 2015), speech comprehension is largely preserved in older adults (Peelle et al. 2010; Wong et al. 2009). Performance, however, declines rapidly in challenging listening conditions, and is accompanied by decreased activation of the auditory cortex (Wong et al. 2009), inferior frontal regions, and reduced connectivity within the speech network (Peelle et al. 2010). Aging individuals tend to engage more working memory and attentional networks (e.g., frontal and prefrontal regions) in a compensatory manner (Wong et al. 2009). Even in the absence of hearing loss, evidence confirms general difficulties in encoding simple and complex sounds, beginning in the brainstem (Clinard and Tremblay 2013) and in the inferior colliculus (Parthasarathy, Herrmann, and Bartlett 2019). Auditory nerve modeling has demonstrated that the deterioration of auditory nerve fibers and loss of inner hair cells impact the brain's capacity to precisely phase‐lock (i.e., align) neural responses to sound onsets (Märcher‐Rørsted et al. 2022), resulting in reduced amplitude and phase coherence of brainstem responses to simple and complex sounds (Clinard and Tremblay 2013). This, in turn, can affect speech processing, as indicated by variable brainstem responses, decreased phase‐locking to speech sounds (Anderson et al. 2012; Bidelman and Alain 2015), and a reduced connectivity between the brainstem and auditory cortex (Bidelman et al. 2019). The weakened sensitivity to auditory input via the brainstem is typically compensated by increased excitability of the auditory cortex (Alain et al. 2014) and altered responses to sounds (Bidelman et al. 2014; Bidelman and Alain 2015; Herrmann, Buckland, and Johnsrude 2019; Herrmann et al. 2016; Herrmann, Maess, and Johnsrude 2023; Ruohonen et al. 2020). Consequently, event‐related potentials (ERPs) recorded by electroencephalography (EEG) exhibit enhanced amplitude responses in aging individuals, particularly in the N100 component (Alain et al. 2014; Bidelman et al. 2014; Brinkmann et al. 2021; Haumann et al. 2023; Herrmann et al. 2016; Ruohonen et al. 2020; Leung et al. 2013). There is consensus in associating these larger ERP responses with the reduced ability to employ “sensory gating” (Brinkmann et al. 2021), an adaptive mechanism to suppress cortical responses to repetitions of predictable stimuli (Ruohonen et al. 2020; Leung et al. 2013). Furthermore, variability in the latency of event‐related responses to sounds (Haumann et al. 2023; Tomé et al. 2015) and the reduction in steady‐state responses to auditory metronomes (Henry et al. 2017; Sauvé et al. 2022) suggest deteriorations in the encoding of the precise timing of sensory events, and in internalizing temporal regularity in auditory sequences. At the same time, there is complementary evidence showing greater neural synchronization with amplitude and frequency modulations of sounds in aging individuals and increased sensitivity to temporal regularities, as revealed by metrics of phase concentration (Herrmann, Buckland, and Johnsrude 2019; Herrmann, Maess, and Johnsrude 2023; Irsik et al. 2021; Parthasarathy, Herrmann, and Bartlett 2019). Larger and less variable event‐related responses (Herrmann et al. 2016) were, however, typically accompanied by reduced sustained neural activity to sound modulations in continuous listening scenario (Herrmann, Buckland, and Johnsrude 2019; Herrmann, Maess, and Johnsrude 2022; Herrmann, Maess, and Johnsrude 2023). These partially contradicting observations leave open the question of whether aging impacts the basic capacities to detect temporal regularities in the sensory environment, generate predictions about the timing of future events, and employ these predictions to optimize sensory processing and perception. In turn, this perspective prompts the question: are difficulties in speech comprehension observed in older adults linked to speech‐specific processing difficulties or to more fundamental temporal processing deficits?

We aimed to investigate whether older adults detect, encode, and employ temporal regularities in the sensory environment to generate predictions and optimize sensory processing similarly to younger adults. We addressed this question by recording EEG in older and younger adults while they listened to isochronous tone sequences and performed a deviant counting task. The task served the scope to focus their attention on the formal properties of the auditory sequences, while diverting their attention from temporal regularity. As such, we did not directly instruct participants to process the timing of sound onsets. We hypothesized that aging would be associated with increased variability in event‐related responses to tone onsets, as indexed by metrics of N100 variability, and reduced Inter‐Trial Phase Coherence (ITPC) in delta‐band oscillatory activity. Furthermore, we hypothesized that older individuals would fail to show a continuous phase‐alignment while listening to isochronous sequences. Finally, we expected a steeper 1/F of the Fourier spectrum in the aging group, indicative of increased cortical excitability. Combined results from event‐related, spectral parametrization, and ITPC analyses confirmed increased cortical excitability and hypersensitivity to sound onsets, and reduced phase coherence in delta‐ and theta‐ frequency bands in the aging group. Altogether, these observations suggest that aging alters basic auditory and temporal processing.

These limitations in fundamental timing capacities in older adults might critically affect not just basic auditory processing but also higher‐order cognitive functions such as speech processing. Consequently, the current results motivate future research on the impact of altered timing capacities on cognition in aging and across the lifespan.

## 2 Materials and Methods

### 2.1 Participants

Forty‐three native German speakers participated in this study and signed written informed consent in accordance with the guidelines of the ethics committee of the University of Leipzig and the declaration of Helsinki. Participants were grouped into 18 younger (HY; 9 females; 21–29 years of age, mean 26.2 years) and 18 older (HO; 9 females; 50–78 years of age, mean 60 years) adults. All participants were right‐handed, had normal or corrected‐to‐normal vision, and no hearing deficits. Participants received 8€/h for taking part in the study. Participants were not asked to indicate musical expertise and/or daily music listening choices.

### 2.2 Experimental Design and Procedure

**FIGURE 1:**
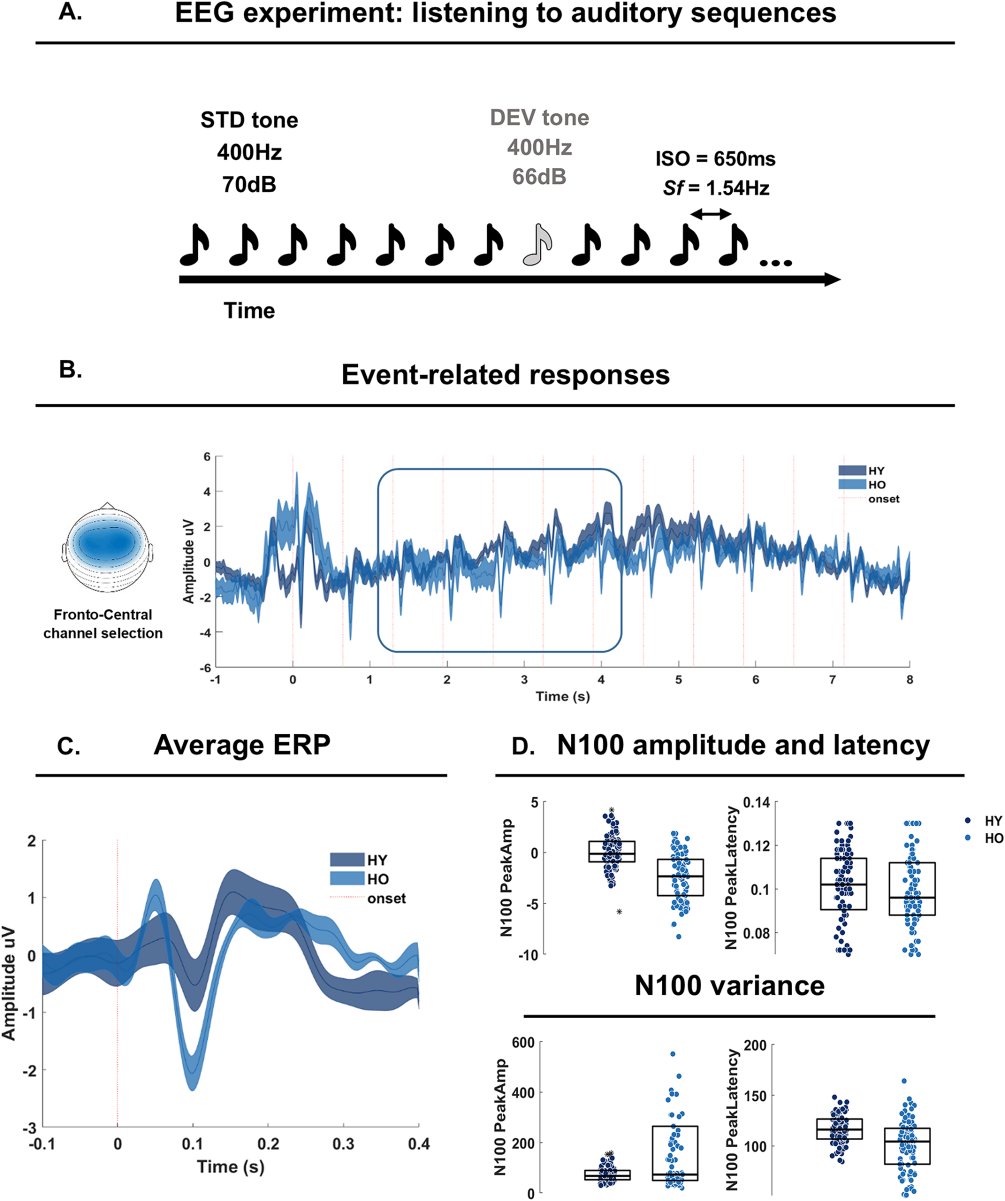
Experimental setup and event‐related analyses. A. Participants listened to 96 isochronous tone sequences, containing 13‐to‐16 tones including standard (STD) tones (*F*0 = 400 Hz, duration = 50 ms, amplitude = 70 dB SPL) and either one or two deviants (DEV; attenuated by 4 dB relative to the STD tones). The first DEV could either fall on Positions 8,9,10, or 11, while the second DEV always fell on Position 12. The inter‐onset‐interval between successive tones was 650 ms, resulting in a stimulation frequency (Sf) of 1.54 Hz. B. Event‐related analyses focused on a fronto‐central (FC) channel cluster (on the left side) and on five tones from the 3rd to the 7th position along the auditory sequence (square on the time‐series). Dark blue lines report the time‐series for younger (HY) participants, while the lighter blue lines report the time‐series for older adults (HO) participants. C. Average ERPs over five tone positions, as highlighted in B, and in the FC channel cluster. Color coding as in B. D. N100 peak amplitude and latency (top panel); one dot per participant, sequence, and tone position. The central horizontal line in each boxplot represents the median, while the edges of the box represent the 25th and 75th percentiles. Stars highlight outliers. At the bottom, the variance in the N100 peak amplitude and latency.

Participants listened to 96 sequences comprising 13‐to‐16 tones (*F*0 = 400 Hz, duration = 50 ms, rise and fall times = 10 ms; amplitude = 70 dB SPL; standard STD), presented binaurally in one recording session of approximately 25 min. Each tone sequence included one or two deviant tones (DEV), attenuated by 4 dB relative to the STD tones, and occurring between the 8th and 12th position. The inter‐onset‐interval between successive tones was 650 ms, resulting in a stimulation frequency (*Sf*) of 1.54 Hz, and a total sequence duration of 8.45–10.4 s (13 to 16 tones * 650 ms; Figure 1A). Participants were seated in a dimly lit soundproof chamber facing a computer screen. Every trial started with a fixation cross (500 ms), followed by an auditory sequence. The cross was continuously displayed on the screen to prevent excessive eye movements while listening to the auditory sequences. At the end of each sequence, a response screen appeared and prompted participants to immediately press a response button to indicate whether they had heard one or two softer tones. After the response, there was an inter‐trial interval of 2000 ms. A session was divided into two blocks of approximately 10 min each, with a short pause in between (about 25 min total duration). Stimulation materials and experimental setups thus mirror those adopted and previously described (Criscuolo, Schwartze, Henry, et al., 2023; Criscuolo et al. 2025).

### 2.3 EEG Recording

The EEG was recorded from 59 Ag/AgCl scalp electrodes (Electro‐Cap International), amplified using a PORTI‐32/MREFA amplifier (DC set to 135 Hz), and digitized at 500 Hz. Electrode impedances were kept below 5kΩ. The left mastoid served as an online reference. Additional vertical and horizontal electrooculograms (EOGs) were recorded.

### 2.4 Data Analysis

#### 2.4.1 EEG Preprocessing

The preprocessing pipeline and the analysis approach adopted here mirror and expand those described previously (Criscuolo, Schwartze, Henry, et al., 2023; Criscuolo, Schwartze, Prado, et al., 2023; Criscuolo et al. 2025). EEG data were analyzed in MATLAB with a combination of custom scripts and functions and the FieldTrip toolbox (Oostenveld et al. 2011). Data were first re‐referenced to the average of the two mastoid electrodes and band‐pass filtered with a 4th order Butterworth filter in the frequency range of 0.1–50 Hz (*ft_preprocessing*). Eye blinks and other artifacts were identified using independent component analysis. This semi‐automated routine combined two steps: in the first iteration, we employed “*fastICA*” (as implemented in FieldTrip) to decompose the original EEG signal into independent components (*N* = number of EEG channels −1), then automatically identified components with a strong correlation (> 0.4; labeled as “bad” components) with the EOG time‐courses, removed them with “*ft_rejectcomponent*”, and then reconstructed the EEG time‐course. In a second step, we again used “*fastICA*” but now with a dimensionality reduction to 20 components. We visually inspected these components via “*ft*_*rejectvisual*”, and selected “outliers” (e.g., based on max values and z‐scores). The 20 components were visually inspected after plotting their topographies and time‐series, and a new selection of “outliers” was defined. Lastly, we visually inspected the two lists of outliers and decided which components had to be removed. On average, we removed two components via “*ft_rejectcomponent*”. Then EEG time‐series were reconstructed. In the next preprocessing step, we performed artifact subspace reconstruction as implemented in the “*pop_clean_rawdata*” function in EEGLAB, and with the “BurstCriterion” parameter set to 20 (all other parameters were set to “off”). We then employed an automatic channel rejection procedure to remove noisy channels. In this routine, we calculated the median variance across channels (and excluding EOG channels), and “outliers” were then defined as exceeding 2.5*median variance. Identified bad channels (max *N* = 2; mean = 1) were interpolated. There were no group differences in the *N* of channels interpolated. Next, we implemented an artifact suppression procedure (Criscuolo, Schwartze, Prado, et al., 2023; Criscuolo et al. 2025), a cleaning routine that interpolates noisy (> absolute mean + 4*S.D) time‐windows on a channel‐by‐channel basis. Lastly, data were low‐pass filtered at 40 Hz via “*ft_preprocessing*”, segmented to each auditory sequence (starting 4 s before the first tone onset and ending 4 s after the last tone onset), and downsampled to 250 Hz.

#### 2.4.2 Event‐Related Analyses

We assessed the amplitude, latency, and variability of neural responses to tone onsets along the auditory sequences by adopting an event‐related potential (ERP) approach. Thus, sequence‐level data as obtained from preprocessing were further segmented into time‐windows ranging from −1 to 8 s relative to the first tone onset in each auditory sequence and later underwent a low‐pass filter with a 20 Hz frequency cutoff (“*ft*_*preprocessing*”). Next, we centered the data by mean correcting each trial by a global average (calculated from −1 to 8 s and across trials) and performed “peak analyses”. Thus, we calculated the participant‐, trial‐, and channel‐level peak amplitude, latency and variability of the N100 component of the ERP (Alain et al. 2014; Bidelman et al. 2014; Brinkmann et al. 2021; Haumann et al. 2023; Herrmann et al. 2016; Ruohonen et al. 2020; Leung et al. 2013). For doing so, we defined a 60 ms‐long time‐windows centered at 100 ms. Within this time‐window we obtained the amplitude peak and its latency (the max value and its time point). Next, we calculated the intra‐individual variability (peak amplitude and latency) across trials and within a fronto‐central channel (FC) cluster of interest. The FC cluster encompassed the sensor‐level correspondents of prefrontal, pre‐, para‐, and post‐central regions highlighted in previous studies (Fujioka et al. 2012) and further highlighted in similar EEG work on rhythm processing (Criscuolo, Schwartze, Prado, et al., 2023; Criscuolo et al. 2025; Nozaradan et al. 2011). The cluster included 16 channels: “AFz”, “AF3”, “AF4”, “F3”, “F4”, “F5”, “F6”, “FCz”, “FC3”, “FC4”, “FC5”, “FC6”, “C1”, “C2”, “C3”, “C4”. As the first tones within an auditory sequence are known to elicit much stronger neural responses compared to later tones, we focused subsequent analyses on tones from the 3rd to the 7th position (STD before the onset of a DEV tone).

##### 2.4.2.1 Statistical Analyses

Statistical analyses assessed group differences in the N100 peak amplitude over tone repetitions along the auditory sequence by means of a repeated‐measure ANOVA. Thus, individual N100 peak amplitudes over five tonal positions (3rd to 7th) were modeled by the “fitrm” algorithm by specifying “Group” and “Time” as factors and allowing for an interaction term. Next, the model entered a repeated measures analysis of variance via the “ranova” function. In the absence of a Group x Time interaction, we proceeded by testing for the main effect of Group.

##### 2.4.2.2 Mixed Effect Models on ERP Data

We assessed group differences in N100 Peak amplitude and latency via Mixed effect models. The model included “Group” as a fixed factor and a random intercept per participant. Model information and results are reported in Table 1 and Table S1, respectively.

**TABLE 1:** Mixed effect model on ERP N100 peak amplitude.

Mixed effect model: ERP N100 peak amplitude							
Model information:							
Number of observations		180					
Fixed effects coefficients		2					
Random effects coefficients		36					
Covariance parameters		2					
Formula:		varOI ~ 1 + Group + (1| participant)					
Model fit statistics							
AIC	BIC	Log likelihood		Deviance			
796.47	809.24	−394.23		788.47			
Fixed effects coefficients (95% CI):							
	Estimate	SE	tStat	DF	*p* value	Lower	Upper
Intercept	0.54	0.81	0.67	178	0.05	−1.06	2.14
Group	−1.66	0.51	−3.24	178	0.001	−2.67	−0.65
Random effects covariance parameters (95% CIs)							
		Type	Estimate	Lower	Upper		
Intercept|participant		Std	1.27	0.91	1.80		
Residual Std		Std	1.93	1.72	2.16		

*Note:* The table reports model information: number of observations, fixed effect coefficients, random effect coefficients, covariance parameters. Then, the formula used to fit the model and model fit statistics: AIC, BIC values, Log Likelihood and Deviance. Further below, the fixed effect coefficients in a 95% confidence interval (CI): estimate, standard error, t‐stat, degrees of freedom (DF), *p*‐value, lower, and upper bound. Right below, random effects covariance parameters: estimate, lower, and upper bound.

##### 2.4.2.3 Statistical Comparisons on the Variance

We assessed group differences in the variability of N100 Peak amplitude and latency by means of permutation testing. This iterative procedure performs 1000 permutations of data points belonging to one or the other group and ultimately assesses the *p*‐value from the original groups against the *p* obtained from permutations. A *p‐*value lower than 0.05 was considered statistically significant. Results are provided in Figure 1D.

#### 2.4.3 Spectral Parametrization

**FIGURE 2:**
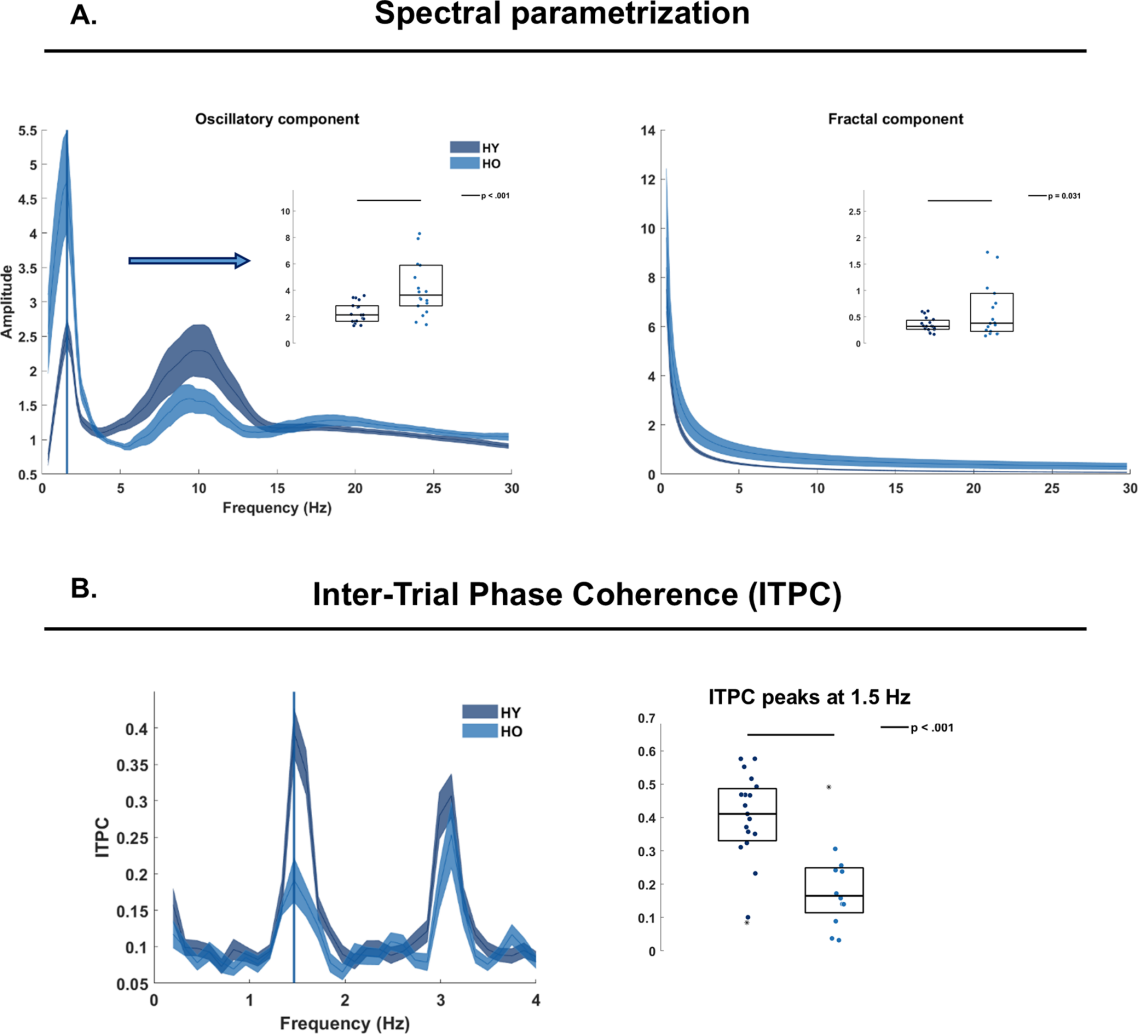
Spectral parametrization and Inter‐trial phase coherence analyses. A. Spectral parametrization analyses allowed to decompose the Fourier spectrum into an oscillatory component (left) and into a non‐oscillatory (fractal) component (right). Both spectra provide frequencies on the x‐axis and amplitude values on the y‐axis. Dark blue lines report the data for younger (HY) participants, while the lighter blue lines report the data for older (HO) participants. The inserts on top of the main panel provide the statistical comparisons on individual data as assessed by permutation testing. On the left, the group comparison assessed the amplitude peak at the stimulation frequency (1.5 Hz). On the right, the group comparison assessed for amplitude differences across the entire spectrum. B. ITPC analyses assessed group differences in the ITPC at the stimulation frequency by means of permutation testing (right). The ITPC spectrum (left) provides frequencies on the x‐axis and coherence values on the y‐axis. The two vertical lines on the spectrum report the group coherence peak.

To investigate how Participants (1) encoded temporal regularities in auditory sequences, (2, 3), and whether there were group differences in the excitation/inhibition balance (Robertson et al. 2019; Voytek et al. 2015), we performed spectral parametrization analyses. Differently from typical Fourier (FFT) analyses, the spectral parametrization allows disentangling oscillatory from non‐oscillatory components (i.e., the 1/F typically observed in the Fourier spectra) (Donoghue et al. 2020). Thus, a series of tone‐locked neural responses should lead to a clear amplitude peak in the frequency spectrum at the *Sf*. To test this hypothesis, we first shortened trials into segments of 8 s (from the first tone onset (0 s) to the 12th tone offset), and then employed the automated spectral parameterization algorithm described in (Donoghue et al. 2020) and implemented in FieldTrip in a two‐step approach. Thus, “*ft*_freqanalysis” was first used in combination with the multi‐taper method for FFT (“mtmfft”) and power as output (“pow”), and secondly by specific “fooof_aperiodic” as output. The output frequency resolution was set at 0.2 Hz. Next, the aperiodic (fractal) spectrum was removed from the FFT spectrum via calling the “*ft*_math” function, finally isolating the so‐called “oscillatory” (Figure 2A, left) from a non‐oscillatory (“fractal”) component (Figure 2A, right).

##### 2.4.3.1 Statistical Analyses

Subsequent statistical analyses were performed on the same FC cluster as described above. Group differences were statistically assessed by permutation testing (1000 permutations) of the extracted peak amplitude values at the *Sf* and the amplitude of the fractal component across the frequency spectrum. A *p‐*value below 0.05 was considered statistically significant.

#### 2.4.4 Inter‐Trial Phase Coherence

When neural activity precisely encodes the temporal regularities in auditory sequences, it should not only show a clear amplitude peak in the FFT spectrum but also display phase coherence. The ITPC metric is inversely proportional to the variability in the imaginary part of the complex FFT spectrum. Thus, when oscillations are precisely aligned over trials (they have the same phase), the ITPC is high; when, instead, there is variability in the phase of the oscillations over trials, the ITPC is lower.

The complex FFT spectrum was obtained by performing FFT decomposition at the single‐participant, −channel and ‐trial level on 8 s‐long segments as above. Next, the ITPC spectrum was calculated by dividing the Fourier coefficients by their absolute values (thus, normalizing the values to be on the unit circle), calculating the mean of these values, and finally taking the absolute value of the complex mean. Further documentation can be found on the FieldTrip website (https://www.fieldtriptoolbox.org/faq/itc/). For illustration purposes, the ITPC spectrum was restricted to 1–4 Hz (Figure 2B).

##### 2.4.4.1 Statistical Analyses

Subsequent statistical analyses were performed on the same FC cluster as for the event‐related analyses. Group differences were statistically assessed by permutation testing of the extracted ITPC values at the *Sf* and with 1000 permutations. A *p‐*value below 0.05 was considered statistically significant.

##### 2.4.4.2 Time‐Resolved ITPC

Although ITPC and similar phase concentration measures are typically interpreted as a proxy of entrainment, they mostly reflect a sequela of time‐locked evoked responses (Breska and Deouell 2017; Haegens and Zion Golumbic 2018; Obleser, Henry, and Lakatos 2017; Zoefel, ten Oever, and Sack 2018). In turn, the variability in the latency of event‐related responses is inversely proportional to the estimated ITPC. As ITPC mostly depends on the (initial) phase estimates from fast‐Fourier transformations, it typically does not allow assessing the dynamics of phase‐alignment in a continuous manner. In other words, we cannot test whether phase coherence changes during the listening period.

Here, we estimated a time‐resolved metric of ITPC (t‐ITPC) to quantify the build‐up of phase‐coherence over the course of an auditory sequence. For doing so, we first employed time‐frequency transform (TF data), then calculated the t‐ITPC using the complex spectra of TF data (same procedure as in the ITPC above) and lastly calculated the slope of the t‐ITPC at the single‐participant level and across frequency bands. More details are provided in the respective paragraphs below.

##### 2.4.4.3 Time‐Frequency Transform

After preprocessing, single‐trial EEG data underwent time‐frequency transformation (“*ft_freqanalysis*”) by means of a wavelet‐transform (Cohen 2014). The bandwidth of interest was centered around the stimulation frequency (± 1 Hz, i.e., 0.54–2.54 Hz, thus obtaining a 1.54 Hz center frequency), using a frequency resolution of 0.2 Hz. The number of fitted cycles was set to three. The single‐trial approach resulted in “induced” (as compared to “evoked”) responses. The output was a complex spectrum; no averaging over channels, trials, or participants was performed at this stage.

##### 2.4.4.4 Slope of t‐ITPC

As for the ITPC above, t‐ITPC was obtained by dividing the complex coefficients of TF‐data by their absolute values (thus, normalizing the values to be on the unit circle), averaging, and finally taking the absolute value of the complex mean. Next, we calculated the slope of each t‐ITPC time‐series by fitting a first‐order polynomial (“*polyfit*” function in MATLAB; p) and then deriving a first‐order approximation (*p*(1)*Time + *p*(2)). The calculation of the slope was performed by starting from the 3rd tone onset and up to the 8th tone (where the DEV tone was likely to occur).

##### 2.4.4.5 Statistical Comparisons

Group differences in the slope of the t‐ITPC at each frequency‐band were assessed via permutation testing and with a total of 1000 permutations.

## 3 Results

### 3.1 Event‐Related Analyses

Event‐related analyses tested for group differences in the N100 component of the event‐related potential (ERP; Figure 2B–D). A repeated‐measures ANOVA tested for group differences in the N100 peak amplitude over five tone positions along the auditory sequences (3rd to 7th position). This analysis specifically assessed a repetition‐suppression effect. The Group * Time interaction term of the model was not significant (*F* = 0.63, *p* = 0.49). We then tested the main effect of Group across the sequence, by pooling the N100 peak amplitudes across the 5 tone positions. The group effect was statistically assessed by mixed effect models and including a fixed effect of group and a random intercept per participant. The model reported a significant group difference in the N100 peak amplitude (t (2,180) = −3.24, *p* = 0.001; Table 1). The mixed effect model testing group differences in the N100 peak amplitude latency was not significant (Table S1).

Next, we assessed group differences in the variability of the N100 peak amplitude and latency. Neither of the two comparisons reported significant group differences.

### 3.2 Spectral Parametrization

After decomposing the Fourier spectrum into a so‐called “oscillatory” component (OSc) and a fractal component (FRc), we statistically assessed group differences in the amplitude of the OSc at the stimulation frequency (1.5 Hz; *Sf*) and the amplitude of the FRc across frequencies by means of permutation testing, and with 1000 permutations. The group effect for the OSc at the *Sf* was statistically significant and showed larger amplitude responses in the HO than the HY (*p* < 0.001; Figure 2A, left). HO also showed a significantly stronger FRc across the spectrum (*p* = 0.031; Figure 2A, right).

### 3.3 Inter‐Trial Phase Coherence

**FIGURE 3:**
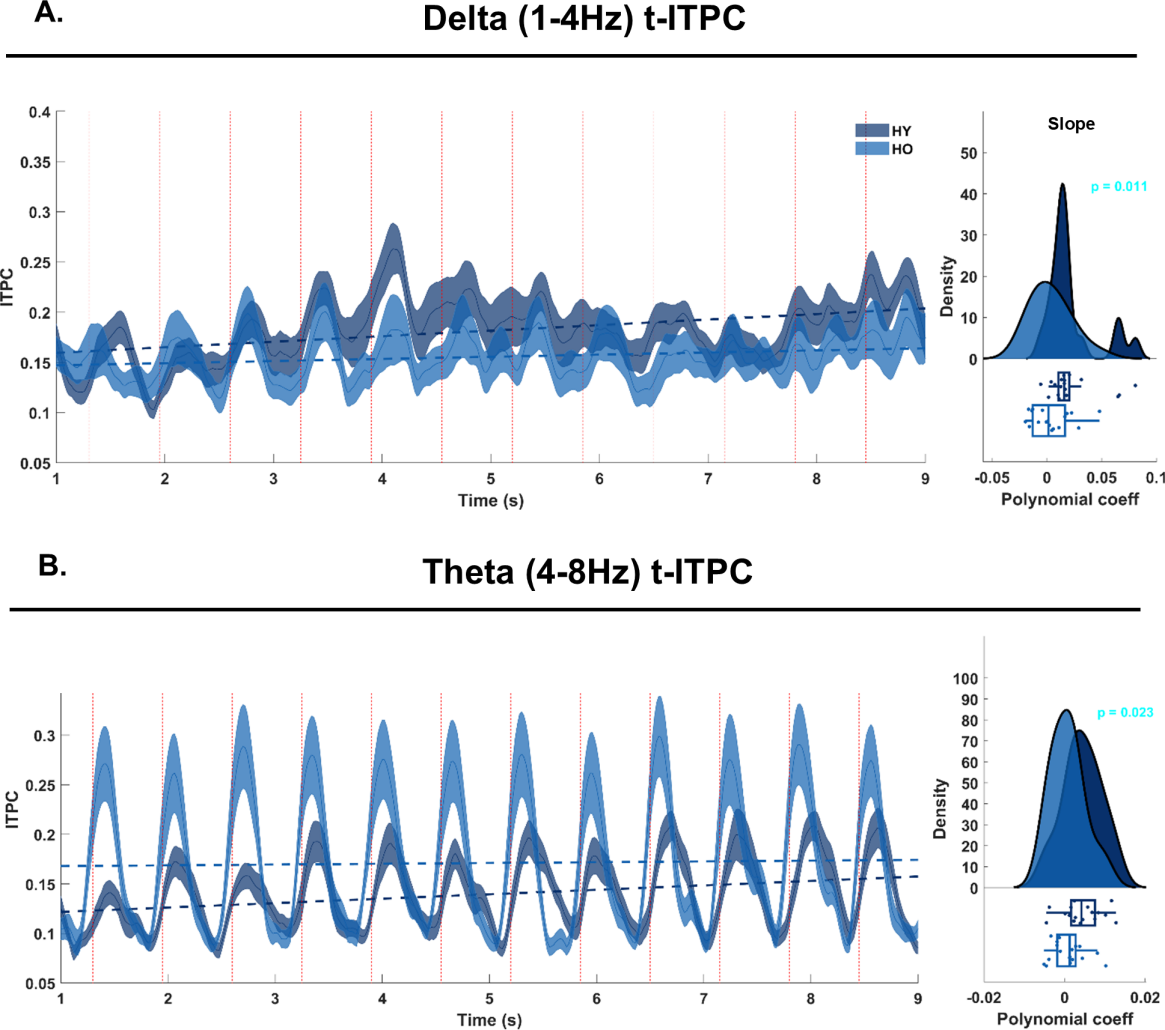
Time‐resolved ITPC. A: the time‐course of the delta‐band time‐resolved inter‐trial phase coherence (t‐ITPC) for HY (darker blue) and HO (lighter blue). Shaded contours report the standard error calculated per group across participants. The x‐axis reports time in seconds, ranging from 1 to 9 s, relative to the onset of the auditory sequence. The y‐axis reports ITPC values. The vertical dotted lines represent tone onsets. The horizontal dotted lines report the slope of the t‐ITPC per group. On the right, the distribution of the slope coefficient across participants is shown, per group. B: the time‐course of theta‐band t‐ITPC for HY and HO as in panel A.

The imaginary part of the complex Fourier spectrum was used to calculate the ITPC Statistical analyses assessed group differences in ITPC at the *Sf*. Permutation testing revealed a significant group effect, with HY showing larger ITPC at the *Sf* as compared to HO (*p* < 0.001; Figure 3B).

### 3.4 Time‐Resolved ITPC

T‐ITPC was obtained by quantifying phase coherence from the complex spectra of continuous wavelet transformed data in the delta‐, theta‐, alpha‐, and beta‐frequency bands. For each participant and frequency band, we calculated the slope of t‐ITPC and later performed group comparisons via permutation testing. HO showed a reduced t‐ITPC slope in the delta (*p* = 0.01; Figure 3A) and theta (*p* = 0.02; Figure 3B) frequency bands, but not in the alpha and beta‐bands.

## 4 Discussion

Throughout the lifespan, neuroanatomical brain changes typically follow an inverted U‐shape trajectory (Gogtay and Thompson 2010). Gray and white matter volume increase from childhood to adulthood and then decline with aging. Concurrently, primary sensory systems often undergo gradual deterioration, potentially leading to decline in auditory processing (Alain et al. 2014; Bidelman et al. 2014; Lai, Alain, and Bidelman 2023). Changes within both peripheral and central auditory systems, such as the deterioration of auditory nerve fibers and loss of inner hair cells, affect the brain's ability to accurately encode sensory events in the auditory environment (Märcher‐Rørsted et al. 2022), consequently impacting speech comprehension, social interactions, and cognition more broadly (Wayne and Johnsrude 2015). Importantly, even in the absence of hearing loss, older individuals experience difficulties in processing both simple and complex auditory sequences in noisy environments (Brinkmann et al. 2021; Clinard and Tremblay 2013; Haumann et al. 2023; Tomé et al. 2015).

In this study, we posited that the challenges observed in higher cognitive processes such as speech processing during aging might stem form an underlying decline in the ability to detect, encode, and employ temporal regularities in the sensory environment to optimize sensory processing. To investigate this hypothesis, we recruited younger and older individuals and recorded their neural activity using EEG while they listened to simple isochronous equitone sequences presented at a stimulation frequency (*Sf*) of 1.5 Hz. Analyses of event‐related potentials (ERP) and spectral data (spectral parametrization analyses) revealed greater evoked responses in older adults, consistent with previous findings documenting hypersensitivity to sensory input in aging (Alain et al. 2014; Bidelman et al. 2014; Brinkmann et al. 2021; Haumann et al. 2023; Herrmann, Buckland, and Johnsrude 2019; Herrmann, Maess, and Johnsrude 2022; Herrmann et al. 2016; Herrmann, Maess, and Johnsrude 2023; Irsik et al. 2021; Ruohonen et al. 2020; Leung et al. 2013). These results support the notion that aging affects the ability to engage in “sensory gating” (Brinkmann et al. 2021), thus failing to ecologically suppress cortical responses to repetitive and predictable stimuli (Herrmann, Buckland, and Johnsrude 2019; Herrmann et al. 2016; Ruohonen et al. 2020; Leung et al. 2013).

Spectral parametrization analyses showed that older adults exhibited an increase in the amplitude of the (1/F) fractal component across frequencies. This observation is consistent with the enhanced event‐related responses described earlier and supports the notion of heightened excitability (or reduced inhibition) of the auditory cortex in aging (Alain et al. 2014). While prior evidence showed diminished encoding of temporal regularity in metronome‐like auditory sequences (obtained through typical Fourier analyses) (Henry et al. 2017; Sauvé et al. 2022), the removal of the fractal component from the frequency spectrum allowed revealing the reversed pattern: enhanced evoked responses to sounds in older individuals. This result seems to confirm prior evidence showing greater neural synchronization with sounds modulations in aging individuals, and an increased sensitivity to temporal regularities (Herrmann, Buckland, and Johnsrude 2019; Herrmann, Maess, and Johnsrude 2023; Irsik et al. 2021; Parthasarathy, Herrmann, and Bartlett 2019). However, the ITPC analyses showed that older adults exhibited lower ITPC at the stimulation frequency (*Sf*; 1.5 Hz), ultimately indicating increased variability in the neural encoding of sound onsets. Thus, we showed that aging is associated with stronger evoked responses, but reduced phase‐alignment. These results support previous observations of reduced coherence and phase alignment of neural activity to sounds in simple and more complex auditory sequences (Anderson et al. 2012; Bidelman and Alain 2015; Clinard and Tremblay 2013; Märcher‐Rørsted et al. 2022).

The hypersensitivity observed in aging individuals is typically further accompanied by reduced sustained neural activity in continuous listening scenario (Herrmann, Buckland, and Johnsrude 2019; Herrmann, Maess, and Johnsrude 2022; Herrmann, Maess, and Johnsrude 2023), potentially affecting speech tracking and comprehension, especially in noisy environments (Herrmann, Maess, and Johnsrude 2022; Irsik et al. 2021). In order words, while older individuals display larger cortical responses to sound onsets, they seem to have difficulties in tracking the temporal fluctuations in the speech envelope (Irsik et al. 2021).

We here asked whether aging individuals would show similar difficulties in tracking and anticipating simple sound onsets in isochronous contexts. To assess the continuous neural dynamics of onset tracking, we employed a time‐resolved ITPC coherence metric and calculated the slope of phase coherence during continuous listening. We showed a flatter slope of t‐ITPC in the delta‐ and theta‐band activity in aging individuals, but no differences in higher frequency bands (alpha and beta). This result confirms a reduction in neural tracking of sound onsets in the aging brain, and further confirms that alpha‐band regulatory mechanisms are intact in older individuals (Herrmann et al. 2023). T‐ITPC analyses also showed increased event‐related phase coherence in theta‐band activity in the HO as compared to HY, potentially indicating the phase‐reset induced by stronger event‐related neural responses to tone onsets.

Taken together, these observations suggest that aging impacts basic auditory and temporal processing. These findings underscore the importance of future research investigating the relationship between basic timing capacities and higher‐order cognitive processes (e.g., speech processing) across the lifespan. Furthermore, findings encourage future research in other sensory modalities: do alterations in basic timing capacities modulate sensory processing across modalities?

This evidence, however, challenges the “exploration‐exploitation shift” hypothesis (Spreng and Turner 2021). According to this perspective, most aging individuals attempt to counteract sensory and cognitive decline by adopting compensatory cognitive strategies and leveraging on previous knowledge predictively (Brown, Gruijters, and Kotz 2022). For example, they may utilize long‐term knowledge, generalizations, and predictions to mitigate increased difficulty with learning and the decline of executive functions due to striatal cholinergic changes (Matamales et al. 2016). To operationalize and test this notion, Brown et al., (Brown, Gruijters, and Kotz 2022) referred to the predictive coding framework: given the reduced certainty of sensory signals, aging individuals rely more on memory and consequently generate predictions about future events (Feldman and Friston 2010). These predictions serve the purpose of adaptation aiming to optimize perception and cognition despite cognitive decline (Moran et al. 2014). Contrary to these expectations, the current findings indicate that older individuals either did not form predictions, or at the very least, did not utilize temporal predictions to inform sensory processing at the fast, millisecond temporal scale. Indeed, consistent with prior electrophysiological evidence, neural responses were not attenuated by top‐down modulatory suppression mechanisms (Alain et al. 2014; Bidelman et al. 2014; Brinkmann et al. 2021; Haumann et al. 2023; Herrmann et al. 2016; Ruohonen et al. 2020; Leung et al. 2013). Alternatively, one may argue that the identified group differences reflect varying levels of attention, motivation, and engagement. While it may be possible to link aging participants' stronger event‐related neural responses to increased levels of attention and motivation as compared to younger participants, the reduction in phase‐alignment metrics clearly point toward alterations in the abilities to adaptively synchronize ongoing brain activity to predictable sound onsets.

The abilities to detect and encode temporal regularities, as well as to form temporal predictions, have been linked to widespread cortico‐subcortical circuitries including the basal ganglia and the cerebellum (Schwartze and Kotz 2013). Lesions in either of these circuitries have been shown to causally impact the ability to predictively align neural dynamics to sound onsets (Criscuolo et al. 2025). Conversely, aging is typically characterized by decreased fractional anisotropy and increased diffusivity (Wassenaar et al. 2019), indicative of white matter deteriorations, along with bilateral gray (Han et al. 2020) and white matter loss in the cerebellum (ce) and reduced connectivity within the dentato‐thalamo‐cortical network (Bernard et al. 2020). Additionally, functional connectivity patterns undergo alterations (Zonneveld et al. 2019), such as reduced within‐network connectivity and variegated patterns of increase and decrease in between‐network connectivity (Grady et al. 2016). Notably, deteriorations within the striatal‐frontal networks (Buckner 2004), the under‐recruitment of the cerebellum during challenging cognitive tasks (Bernard et al. 2020), and changes within the cerebellum‐basal ganglia circuitries (Hausman et al. 2020) have been associated with reduced cognitive control (Spreng and Turner 2021) and numerous motor and cognitive deficits (Hausman et al. 2020).

Aligned with the original hypotheses and bolstered by these novel findings, we propose that neuroanatomical and functional alterations in cortico‐subcortical circuitries, including the basal ganglia (BG) and the cerebellum (ce) may impact fundamental timing and predictive abilities, which are integral to cognition. These changes could impact the documented declines in processing speed, working memory, inhibition, memory, and reasoning capacities (Cabeza et al. 2018; Reuter‐Lorenz and Park 2014; Salthouse 2019). However, establishing a causal link between timing, predictive functions, and general cognition presents a challenge due to the substantial heterogeneity in aging trajectories. Indeed, neuroanatomical and cognitive changes throughout the life are subject to modulation by a complex interplay of vascular, metabolic, and inflammatory risk factors (Raz and Daugherty 2018), which, in turn, are influenced by the intricate interaction of environmental factors (e.g., socioeconomic status and education) and genetic predispositions (Cabeza et al. 2018). Variability in any of these modulating variables inevitably results in significant diversity in cognitive capacities among older adults, impeding generalizations. This observation may be particularly important for the current aging sample spanning a range of 28 years. This could have potentially increased interindividual variability in the background modulating factors, as well as in attention capacities and motivation. Another limitation of the current study could be the lack of systematic audiometry testing in our aging population. Although participants did not report any hearing deficits, it remains possible that varying levels of hearing in our sample may lead to differences in neural responses to sounds, thus limiting the interpretations and generalizability of results.

Therefore, systematic, longitudinal, and comprehensive assessments of timing and cognitive functions across the lifespan are imperative to further elucidate the link between cognition and basic timing processes.

## 5 Conclusions

Here, we examined the effects of aging on basic sensory and temporal processing. The integration of findings from three complementary analytical methods highlights the adverse effects of aging on the fundamental capacities to encode the timing of sound onsets in continuous streams and suppress cortical responses to predictable stimuli. This evidence motivates future research on the link between basic timing functions and general cognition, across the lifespan.

## Author Contributions

S.A.K., M.S., conceptualized the study. S.A.K., M.S., collected the data. A.C. designed and performed data analyses. A.C., S.A.K., M.S., L.B. interpreted the results. A.C. wrote the first draft of manuscript. All authors contributed to, revised, and approved the final version of the manuscript.

## Conflicts of Interest

The authors declare no conflicts of interests.

### Peer Review

The peer review history for this article is available at https://www.webofscience.com/api/gateway/wos/peer‐review/10.1111/ejn.70031.

## Data Availability

The analysis code and data in use here can be provided upon reasonable request by the corresponding author.
